# Status epilepticus and coma leading to death in a boy caused by Medium-chainacyl-coA dehydrogenase deficiency

**DOI:** 10.22037/ijcn.v15i4.23925

**Published:** 2021

**Authors:** Ezatolah ABBASI, Ahad GHAZAVI, Masoud HASSANVAND AMOUZADEH, Mohammad VALIZADEH, Mohsen AKHAVAN SEPAHI

**Affiliations:** 1 Pediatric Neurologist, Urmia University of Medical Sciences, Urmia, Iran.; 2 Neuroscience Research Center, Qom University of Medical Sciences, Qom, Iran; 3 Pediatrician, Urmia University of Medical Sciences, Urmia, Iran; 4 Department of Pediatric Nephrology, Qom University of Medical Sciences, Qom, Iran

**Keywords:** Hypoglycemia, Medium-Chain Acyl-coA Dehydrogenase Deficiency (MCADD), Metabolic screening

## Abstract

Medium-chain acyl-coA dehydrogenase deficiency (MCADD) is an autosomal recessive disorder of fatty acid β- oxidation, which is inherited in an autosomal recessive manner. The enzyme plays a role in hepatic Ketogenesis, which is a significant source of energy during prolonged fasting. There is no metabolic screening program except for phenylketonuria (PKU) and hypothyroidism in Iran, and such screening is exclusively implemented in the case of babies with unprovoked seizures and hypoglycemia and previous unexplained sibling deaths. In this paper, we report a case of a seven-year-old boy who presented with afebrile serial seizures leading to coma and death. IN this regard, metabolic screening tests were used to determine the exact cause of encephalopathy and the final diagnosis.

## Introduction

Medium-chainacyl-coA dehydrogenase deficiency (MCADD) is an autosomal recessive inborn error of mitochondrial fatty acid β-oxidation (1). It is also the most common inherited disorder of mitochondrial fatty acid oxidation. The early detection of MCADD in neonatal screening programs provide such patients with better outcomes as the parents receive some instructions to avoid prolonged fasting and use high carbohydrate content dishes during intercurrent illnesses (7).

There have been about 20 case reports in Asia (2). In Iran, because of the lack of a routine metabolic screening program, the samples are delivered to a laboratory in Germany in the case of high clinical suspicion. Herein we describe a seven-year-old boy, who suddenly developed serial afebrile seizures with no history of developmental delay and previous seizures. The seizures were followed by the loss of consciousness and finally lead to death in two days.

Considering the death of his older sibling with similar clinical presentation and detecting hypoglycemia without ketosis in the urine sample, we took dry blood spot samples for tandem mass spectrometry. The results sent by the lab confirmed the MCADD diagnosis. Introducing neonatal metabolic screening programs may contribute to avoiding similar scenarios.

## Case Report

The patient was a 7-year old boy from Miandoab, a city in the south of West Azarbaijan province, Iran. He exhibited no history of prenatal and postnatal diseases and had a normal growth. His healthy parents were cousins. He had successfully passed the first grade of the primary school. The patient suddenly experienced febrile serial generalized tonic-clonic seizures and the hospitalized in Shahid Abbasi teaching Hospital in Miandoab.

After controlling his seizures using the bolus doses of phenobarbital and phenytoin, his consciousness level decreased, thereby necessitating endotracheal intubation and assisted ventilation. Then the patient was transferred to our tertiary subspecialty ward in Urmia’s Motahari Hospital. When we visited the patient for the first time, he was suffering from a deep coma; however, his vital signs were normal.

On physical examinations, his pupils were dilated with inadequate response to light, the liver was palpable about 4cm below the costal margin; his muscular tone severely diminished, and deep tendon reflexes were undetectable. His examination was otherwise normal.

Routine laboratory tests revealed a nonketotic hypoglycemia (Table 1) and elevated liver enzymes (Table 2). According to the endocrinology consultation, a blood and urine sample was obtained to further evaluation into the exact cause of hypoglycemia. The obtained results ruled out ethiologies such as lipid malabsorbtion (TG=92 mg/dl¸ cholesterol 73 mg/dl¸ LDL=37 mg/dl; disorder of respiratory chain (lactate =13 ng/dl)¸ adrenal insufficiency ( cortisol=62.4µg/dl)¸ hypopituitarism (ACTH=411 pg/ml), and hyper insulinemia (insulin= 0.7µIU/ml)[Figure 3]

.

Laboratory tests suggested by our pediatric gastroenterologist to detect the viral or immune cause of hepatitis revealed nothing, and the urine toxicology screening test was negative. Since his older male sibling died five years ago with the similar symptoms, the inherited inborn errors of metabolism were highly likely; thus, we delivered dried blood spot samples to a lab in Germany for tandem mass spectrometry. The brain computerized tomography revealed no edema. Unfortunately, the patient died two days after admission due to multiple organ system failures. The acylcarnitine analysis showed significantly elevated levels of medium-chain acylcarnitines (hexanoylcarnitine(c6)1.3µmol/lit (0- 0.15) and octanoylcarnitine(c8)0.73 µmol/lit (0- 0.23), which is compatible with medium-chain acyl-coA dehydrogenase deficiency (Table 4).

The filter paper screening also revealed no indication of congenital hypothyroidism ¸ adrenal hyperplasia¸ galactosemia ¸biotinidase deficiency¸ amino acid metabolism disorders and tyrosinemia typ1. Molecular genetics verification was impossible due to the patient's death.

## Discussion

Medium-chainacyl-coA dehydrogenase is a mitochondrial enzyme catalyzing the dehydrogenation of acyl coA with a chain length of 4-12 carbon atoms. Lack of the MCAD enzyme makes glycine, carnitine esters, and dicarboxylic acids accumulate within the body, thereby arousing specific disease presentation with hypoglycemia, vomiting, and encephalopathy during an intercurrent illness or prolonged fasting (1-4). Hepatomegaly and liver damage are often associated with rapid development of coma and death; hence, 16% of the survived cases would suffer from severe neurologic deficits (4,5). The acylcarnitine profile of patients with MCADD is characterized by a high level of C6-C10 species, especially octanoylcarnitine (1, 3, 4, 6).

Although there is a metabolic screen test for the diagnosis and screening of patients with MCADD, given the likelihood of the diseases reoccurrence in subsequent children, adopting some measures seems necessary to prevent the recurrence of the same diseases and make a diagnosis before birth by using genetic evaluation and detecting gene mutation as Homozygote and Heterozygote for the 985A>G (4, 7, 8, 9).

This patient presented with status epilepticus, coma, hepatomegaly, and impaired liver function tests died in a few days. To sum up, we faced a patient suffering from hepatic encephalopathy, and differential diagnosis included viral, immune, toxicologic, and metabolic factors. Our investigation of hepatotropic and non-hepatotropic viruses revealed nothing; however, the toxicology screening tests and immunologic assessments introduced metabolic causes as the main factor, given the history of his older sibling's death with similar clinical presentations. Accordingly, we performed a metabolic screening test to rule out respiratory chain disorders, tyrosinemia type 1, Hashimoto encephalopathy, galactosemia, the metabolism disorders of amino acids, and fatty acid oxidation chain disorders. Acylcarnitine analysis showed significantly-elevated levels of medium-chain acylcarnitines (C6_C10 species), indicating the MCAD deficiency as the cause of hepatic encephalopathy. We failed to justify the diagnosis based on molecular genetic testing because of the death of the patient (7,10).

A metabolic screening program might contribute to the early detection and treatment of metabolic disorders (8, 11). Similarly, molecular genetic testing would facilitate providing prenatal diagnosis and treatment as well as reproductive counseling to the involved families.

**Table 1:** Biochemistry

TEST	Result	Unit	Reference value
BUN	37	Mg/dl	7-20
Creatinin	0.6	Mg/dl	0.5-1.3
AST	234(H)	u/L	5-40
ALT	86(H)	u/L	5-40
ALKL-P	371	Iu/L	180-1200
Blood sugar	<20	Mg/dl	
Serum Na	136	MEq/l	135-148
Serum K	5.1	MEq/l	3.5-5
Serum Ca	8.3	Mg/dl	8.6-10
CRP	19		<10 negative >10 positive
WBC	37	Mg/dl	7-20
Hb	0.6	Mg/dl	0.5-1.3
HCT	234(H)	u/L	5-40
PLT	86(H)	u/L	5-40
ESR	371	Iu/L	180-1200
BUN	37	Mg/dl	7-20
Creatinin	0.6	Mg/dl	0.5-1.3
AST	234(H)	u/L	5-40
ALT	86(H)	u/L	5-40
ALKL-P	371	Iu/L	180-1200
Blood sugar	<20	Mg/dl	
Serum Na	136	MEq/l	135-148
Serum K	5.1	MEq/l	3.5-5
Serum Ca	8.3	Mg/dl	8.6-10
CRP	19		<10 negative >10 positive

**Table 2:** Biochemistry

TEST	Result	Unit	Reference value
Ceruloplasmin	0.230	g/d	0.15-0.30
LKM	1.6	IU/ML	<20 negative >20 positive
ASMA	<1:100	titer	< 1:100 negative
Alpha1 Antitrypsin	1.56	g/d	0.9-2
CPK	19257 (H)	Iu/L	20-190
LDH	2550 (H)	Iu/L	200-450

**Table 3:** Biochemistry & Hormones

TEST	Result	Unit	Reference value
Uric acid	14.9	mg/dl	3-7
Cholesterol	73	mg/dl	normal<200
Triglyceride	92	Mg/dl	normal<200
HDL	22	Mg/dl	30-80
Lactate	13	Mg/dl	Normal 4.5-20 Mg/dl
Ammoniac	187	Mg/dl	19-90
T3	35	Ng/dl	76.3-220
TSH	0.14	Miu/ml	0.30-5.60
Cortisol	62.4	Microgram/dl	Child 3.0-21.0
Insulin	0.7	Miu/ml	3.21-16.3
ACTH	411	Pg/ml	1.4-106

**Table 4:** Acyl carnitine analysis

	Result	Reference Range
Hexanoylcarnitine(c6)	1.03µmol/lit	<0.40 µmol/lit
Octanoyl carnitine(c8)	0.73 µmol/lit	<0.20 µmol/lit
Decanoyl carnitine(c10)	0.26 µmol/lit	<0.40 µmol/lit
Decenoyl carnitine(c10:1)	0.69 µmol/lit	<0.20 µmol/lit

Acyl carnitine analysis showed significantly elevated levels of Medium-chain acylcarnitines; hence, Medium-chain acyl-coA dehydrogenase deficiency (MCADD) must be suspected.
